# Determinants of clinical outcome and length of stay in acute care forensic psychiatry units

**DOI:** 10.1186/s12888-023-04748-2

**Published:** 2023-04-18

**Authors:** Isabella D’Orta, Kerstin Weber, François R. Herrmann, Panteleimon Giannakopoulos

**Affiliations:** 1 Division of Institutional Measures, Medical Direction, Geneva University Hospitals, Geneva, Switzerland; 2 Department of Rehabilitation and Geriatrics, Geneva University Hospitals and University of Geneva, Geneva, Switzerland; 3 Department of Psychiatry, University of Geneva, Geneva, Switzerland; 4 Institute of Global Health, University of Geneva, Geneva, Switzerland

**Keywords:** Acute psychiatric care, Clinical evolution, Detained persons, Prison, Psychiatry

## Abstract

Criminological and sociodemographic variables, such as previous criminal convictions, increased risk of violence, early onset of mental disorder, antisocial personality, psychosis and low social support, have all been related to longer length of stay (LoS) and poorer outcome in long stay forensic services. The factors impacting on LoS and clinical response in acute care specialized units are poorly documented. To address this issue, we examined the psychiatric records of all cases admitted between January 1st and December 31th 2020 in the sole acute ward for detained persons located in the central prison of the Geneva County, Switzerland. Information on judicial status included pre-trial versus sentence execution, previous incarcerations, and age of the first incarceration. Sociodemographic data included age, gender, marital status, and education attainment. Previous inpatient stays prior to incarceration were recorded. All of the ICD-10 clinical diagnoses were made by two independent, board-certified psychiatrists blind to the scope of the study. The standardized assessment was based on the HoNOS (Health of Nation Outcome Scales) at admission and discharge, HONOS-secure at admission, HCR-20 (Historical Clinical Risk 20) version 2, PCL-R (Psychopathy Checklist Revised), and SAPROF (Structured Assessment of Protective Factors). Stepwise forward multiple linear regression models predicting the LoS and delta HONOS respectively were built with the above mentioned parameters. The selected variables were then used in univariate and multivariable regression models. Higher HCR-scores (mainly on clinical items), and longer LoS were related to higher delta HONOS scores. In contrast, cases in pre-trial detention showed a worst clinical outcome. In multivariable models, all three variables remained independent predictors of the clinical outcome and explained 30.7% of its variance. Only education and diagnosis of borderline personality were related to the LoS and explained 12.6% of its variance in multivariable models. Our results suggest that the use of acute wards specialized in forensic psychiatry are mainly useful for patients with prior inpatient care experience, and higher violence risk during sentence execution. In contrast, they seem to be less performant for persons in pre-trial detention that could benefit from less restrictive clinical settings.

## Introduction

The number of forensic mental health services increased steadily in many Western countries from early 90’s as a consequence of a variety of factors such as the significant burden of acute and long-standing psychiatric conditions among detained persons, decrease of capacity in general psychiatry but also expansion in the types of psychiatric defences in courts of law and public concerns about violent behaviour attributed to the mentally ill [1, 2]. Short stay forensic services are designed to admit inmates displaying acute symptoms associated with self or others-threatening behaviour and need for urgent psychiatric care [1–3]. Long stay services allow for forensic inpatient treatment, referred to as “psychiatric detention”, “protective” or court ordered treatment (COT), which often exceeds the maximum length of a prison sentence that would be adjudicated for similar offenses committed by healthy perpetrators [4]. In this latter case, the treatment requires extended time since it focuses on both mental health improvement and ensuring public safety. A main concern regarding these units is their efficiency since they are usually high-cost and low-volume services. Moreover, disproportionately long and protracted stays in forensic institutions can lead to human right violations but premature discharge of unstable patients may be equally deleterious leading to worse overall outcomes, poorer quality of life, and increased violence and re-admission risk [5].

Several studies addressed the determinants of length of stay (LoS) and treatment outcome in long stay forensic services with some convergent observations but also conflicting data. These parameters may depend on the course of disease and severity of symptoms, compliance with previous treatments, family support, but also criminological characteristics. Previous criminal convictions, increased Historical Clinical Risk (HCR)-20 risk item scores, violent crimes, younger age, low social support and presence of pervasive psychotic symptoms (including diagnosis of schizophrenia and schizoaffective disorder), treatment resistance, as well as previous contacts with child and adolescent psychiatric services have been consistently related to longer LoS and poorer outcome. Among the other diagnoses, substance use disorders and cluster B personality disorders were associated with worst clinical outcome, yet their impact on LoS remains less clear [6–12]. In addition, external factors related to the judicial system, criteria for admission, and allocation of resources may also impact on both LoS and clinical outcome [13]. The determinants of clinical outcome in the particular case of high and medium security hospitals are less well established. Most previous studies focused solely on criminal recidivism and pointed to well known risk factors such as procriminal companions, attitudes/cognitions supportive of criminal behavior, antisocial personality but also young age at first crime, early onset of mental disorder, and previous forensic treatments. In contrast, clinical variables (except antisocial personality) did not have much predictive value [2, 3, 14–16].

In contrast to the long stay forensic services, the determinants of LoS and clinical response in short stay forensic units are rarely addressed. These units play a key role in detention since they have to manage disruptive behaviours and suicidality of inmates that may represent an acute reaction to incarceration or expression of long-lasting vulnerability. It is thus crucial to assess the evolution and outcome of patients treated in these services, both to ensure effectiveness and quality of care and define the profiles of users who may benefit from specialized acute care in forensic settings [17, 18]. The differences between the detained individuals treated in short stay forensic units compared to patients admitted in acute care units of general psychiatry are still matter of debate. From some authors, the presence of a legal framework implies an artificial distinction between the two populations [3, 19, 20]. However, other analyses showed significant differences in the demographic and clinical profile of detained persons admitted in inpatient forensic units in high and medium-security hospitals compared to patients treated in general psychiatry [21–24]. They are more often single, with higher suicide risk [21], more frequent psychotic beliefs [22], lower education attainment and occupational levels [23, 24]. The present study explores the determinants of LoS and clinical outcome in detained persons admitted to an acute care secure ward located in the central prison of Geneva, Switzerland. Our *a priori* hypothesis was that environmental factors (pre-trial versus sentence execution), criminological variables (HCR-20 risk score) and psychiatric diagnosis (personality disorders, schizophrenia, substance use disorders) impact on the duration and outcome of the clinical stay. We also postulated that this would not be the case for parameters usually affecting long term prognosis such as young age at first crime and previous criminal convictions.

## Materials and methods

### Subjects

The UHPP (Unité hospitalière de psychiatrie pénitentiaire) is a 15 bed unit specially designed for acute psychiatric care of detained persons from the French speaking counties and is part of a medium-security hospital located in prison. Admission to the UHPP was based on need for urgent psychiatric care because of the presence of acute depressive or psychotic symptoms, psychomotor agitation with self or others-threatening behaviours.

The health care team is composed of 4 medical doctors, 35 nurses and one nurse-auxiliary. 5 nurses are present during every day shift (2 between 9 pm and 7 am). Prison staff is continuously present, usually 2 to 4 prison guards for shift. They guarantee the security during daily activities in the unit.

Care programs are based on the integration of psychopharmacology and psychotherapeutic approaches. The vast majority of the patients receive psychotropic medication. They also systematically benefit from at least one clinical encounter with a nurse during the day and 4 to 5 clinical encounters with the medical doctors during the week. Group therapy, art-therapy and ergotherapy (a physical therapy aiming to reduce pain, discomfort and functional disability) are performed on a regular basis. Clinical activities take place respecting the prison timetable/schedule. Patients are allowed to spend time together in a common room (two hours in the morning and three hours in the afternoon). Following the penitentiary international rules, patients are allowed to spend one hour of time in the yard of the unit, where a tennis table is available. Five slots per day are scheduled for smoking patients, during which they have access to a limited part of the yard.

We examined the psychiatric records of all cases admitted between January 1st and December 31th 2020 (total number of admissions = 261). All of the cases with duration of stay less than 10 days (n = 31) were excluded. In these cases, the hospital stay was interrupted because of the rapid improvement of the symptoms and formal request to return in prison or by court decision, not allowing for obtaining the information needed for clinical and forensic assessment. Multiple admissions were registered in 150 cases whereas the remaining 80 cases were admitted only once during the period of reference. To prevent overrepresentation of those repeatedly admitted, we randomly selected 30 cases for each group (repeated and single admissions). The final sample included 60 cases (mean age: 34.8 ± 11, mean LoS 24.8 ± 29.2).

Each patient was assigned an identification number that was derived from the name and birth date and subsequently encrypted. Information on judicial status included pre-trial versus sentence execution, previous incarcerations, and age of the first incarceration. Sociodemographic data included age, gender, marital status, and education attainment. Psychiatric history including inpatient stays prior to incarceration were recorded. All of the ICD-10 clinical diagnoses were made at the time of admission by two independent, board-certified psychiatrists (during the hospital stay and after discharge), blind to the scope of the study. Only cases with concordant psychiatric diagnoses (the two independent diagnoses should be identical) were considered in this sample. Psychiatric diagnoses included adjustment disorders, bipolar disorder, depressive disorders (ICD-10 codes F32-33), personality disorders (antisocial and borderline disorders), psychosis (ICD-10 codes F20-F29) and intellectual disability. Presence of suicidal behaviour and substance use disorders were treated as binary variables.

### Assessment tools

The HoNOS (Health of the Nation Outcome scale) is one of the most used clinical outcome measure. It is a questionnaire of 12 scales covering areas of problems experienced by working-age adults in contact with specialised mental health services. It is systematically performed at the admission and at the discharge of every patient. This instrument covers domains of behaviour, impairment, symptoms and social functioning [25]. We also used at admission the HoNOS-S (Health of Nation Outcome Scales-Secure), a measure that includes both clinical and security scales specially designed to assess the needs of individuals experiencing mental illness who have offended. It comprises seven security items added to the traditional 12 HoNOS items security scale. The security items include risk of harm to adults or children, risk of self-harm, need for building security to prevent escape, need for a safely staffed living environment, need for escort on leave (beyond secure perimeter), risk to individual from others, and need for risk management procedures [26].

HCR-20 (Historical Clinical Risk 20) version 2: it is composed of one static and two dynamic clinician reported scales. This scale is a wide range violence assessment tool developed by Webster, Evan, Douglas, and Witrup in 1995 using a sample of institutionalized people who were followed for approximately 2 years after their discharge into the community [27]. HCR-20 consists of 20 items and assesses past, present and future indicators of violence [28, 29]. Following Brunero & Lamont [30] it is the most widely used risk assessment tools. It contains 20 items: 10 related to historic items (previous violence, age at first violent incident, relationship instability, employment problems, substance use problems, major mental illness, psychopathology, early maladjustment, personality disorder and prior supervision failure [31]; five clinical items (lack of insight, negative attitudes, active symptoms of major mental illness, impulsivity and unresponsiveness to treatment); and five ‘risk management’ items (plans lack feasibility, exposure to destabilizes, lack of personal support, non-compliance with remediation attempts and stress) [32].

PCL-R (Psychopathy Checklist Revised): It is a pivotal tool to identify psychopathic individuals in correctional settings, [33, 34]. In 1991 Hare designed this scale to measure the clinical construct of psychopathy, and since it has become the leading instrument to predict recidivism, violence and treatment outcome [35–37].

SAPROF (Structured Assessment of Protective Factors): it is a scale designed as a complement to assess risk considering protective factors. The SAPROF items are classed into three areas: internal, motivational, and external factors. Items 1 and 2 (internal factors) are considered static, whereas the other 15 factors are dynamic and therefore likely to change during treatment [38].

The criminological assessment was routinely available for all of the cases admitted for the first time to the UHPP irrespective of their judicial status (pre-trial versus sentenced).

### Statistical analysis

Fisher exact, unpaired Student t and Mann-Whitney u tests were used to compare sociodemographic (age, gender, marital status, education), clinical (psychiatric inpatient care prior to incarceration, suicidal behavior, psychiatric diagnosis, substance use disorders), outcome (length of stay and delta HONOS-S (admission-discharge)) and criminological (PCL-R, HCR-20 and its items, SAPROF scores) variables between pre-trial and sentence execution cases. Marital status (married, separated-divorced, single), education and presence of previous inpatient care were treated as ordinal variables. Suicidal behaviour was treated as binary variables. Psychiatric diagnoses included adjustment disorders (ICD 10 code F43), bipolar disorder (ICD 10 codes F30-F31), depressive disorders (ICD-10 codes F32-33), personality disorders (ICD 10 codes for antisocial and borderline personality), anxiety disorders (ICD 10 code F40-F42) and psychosis (ICD-10 codes F20-F29). Correction for multiple comparison in Table 1 was performed using the Benjamini-Hochberg method. Cases with multiple diagnoses were considered in each diagnostic group separately. Stepwise forward multiple linear regression models predicting the logarithm of LoS (to obtain normally distributed data) and delta HONOS respectively were built with all of the above mentioned variables (included in Table 1). The selected variables were then used in univariate and multivariable regression models. The significance level was set at P < 0.05. All statistical analyses were performed using Stata 17.0.

**Table 1:** Demographic, clinical and criminological characteristics in the present series. P value threshold according to Benjamini-Hochberg = 0.01087

	PRETRIAL DETENTION				
	No	Yes	Total	Pvalue	
N	29	31	60		
Age	36.4 ± 11.2	33.3 ± 10.8	34.8 ± 11.0	0.270	
Length of stay	19.2 ± 13.7	30.0 ± 38.0	24.8 ± 29.2	0.146	
DELTA HONOS	10.3 ± 5.1	7.5 ± 4.1	8.9 ± 4.8	0.021	
**HONOS-S**	32.7 ± 13.4	21.8 ± 8.2	27.1 ± 12.2	< 0.001	*
**PCL-R**	14.8 ± 7.2	7.9 ± 6.6	11.2 ± 7.6	< 0.001	*
HCR-20	19.8 ± 7.7	16.9 ± 7.7	18.3 ± 7.8	0.153	
Historical	10.5 ± 4.2	7.4 ± 4.3	8.9 ± 4.5	0.007	*
Clinical	4.6 ± 2.6	4.0 ± 2.7	4.3 ± 2.7	0.376	
Risk	4.3 ± 2.0	5.2 ± 2.3	4.8 ± 2.2	0.137	
**SAPROF**	15.6 ± 6.6	10.6 ± 7.6	13.0 ± 7.5	0.009	*
**Previous incarcerations**				< 0.001	*
First	4 (13.8%)	19 (61.3%)	23 (38.4%)		
Multiple	24 (82.8%)	8 (25.8%)	32 (53.3%)		
Unknown	1 (3.4%)	4 (12.9%)	5 (8.3%)		
Age at first incarceration	33.3 ± 9.1	32.9 ± 10.3	33.1 ± 9.7	0.779	
Education				0.826	
Apprenticeship	4 (13.8%)	5 (16.1%)	9 (15.0%)		
Primary	19 (65.5%)	20 (64.5%)	39 (65.0%)		
Secondary	3 (10.3%)	4 (12.9%)	7 (11.7%)		
Specialized	3 (10.3%)	0 (0.0%)	3 (5.0%)		
University	0 (0.0%)	2 (6.5%)	2 (3.3%)		
Marital status				0.089	
Separed-divorced-widowed	1 (3.4%)	3 (9.7%)	4 (6.7%)		
Married	4 (13.8%)	9 (29.0%)	13 (21.7%)		
Single	24 (82.8%)	19 (61.3%)	43 (71.7%)		
Previous care				0.091	
No history	2 (6.9%)	7 (22.6%)	9 (15.0%)		
Unknown	1 (3.4%)	4 (12.9%)	5 (8.3%)		
Yes	26 (89.7%)	20 (64.5%)	46 (76.7%)		
Gender male	22 (75.9%)	27 (87.1%)	49 (81.7%)	0.327	
Substance use disorder	20 (69.0%)	22 (71.0%)	42 (70.0%)	1.000	
Suicidal behavior	8 (27.6%)	13 (41.9%)	21 (35.0%)	0.287	
Adjustment disorder	3 (10.3%)	6 (19.4%)	9 (15.0%)	0.474	
Antisocial personality	3 (10.3%)	0 (0.0%)	3 (5.0%)	0.107	
Bipolar disorder	1 (3.4%)	2 (6.5%)	3 (5.0%)	1.000	
Borderline personality	6 (20.7%)	6 (19.4%)	12 (20.0%)	1.000	
Mood disorders	2 (6.9%)	7 (22.6%)	9 (15.0%)	0.148	
Psychotic disorders	19 (65.5%)	11 (35.5%)	30 (50.0%)	0.038	

## Results

Cases in pre-trial detention displayed significantly lower HONOS-S scores at baseline, delta HONOS-S scores, as well as PCL-R and SAPROF scores compared to those in sentence execution. Interestingly, the percentage of cases with psychotic disorders was significantly higher in sentence execution yet this differences did not survive after correction for multiple comparisons (Table 1).

The stepwise forward multiple linear regression models identified six candidate variables explaining the delta HONOS-S score: education, male gender, HCR-20, LoS, number of previous inpatient stays, and pre-trial detention. In univariate models, four among them were significantly associated with the clinical outcome. Higher HCR-scores (historical and clinical items), longer LoS and number of previous inpatient stays were related to higher delta HONOS-S scores. In contrast, cases in pre-trial detention showed a worst clinical outcome with lower differences in HONOS-S scores between admission and discharge. In multivariable models, clinical item score of the HCR-20, longer LoS and pre-trail detention were the more significant predictors of the clinical outcome and explained as much as 30.7% of its variance (Table 2).

**Table 2:** Results of univariate (Coeff. unadjusted) and multiple (Coeff. adjusted OR) linear regression associated with the Delta HONOS. N = 60. R^2^ : coefficient of determination; R^2^ adj: adjusted R^2^. Model 1 (total HCR-20 scores): R^2^ = 0.354; R^2^ adj = 0.252. Model 2 (HCR-20 item scores): R^2^ = 0.413; R^2^ adj = 0. 307

							Model 1				Model 2							
Characteristics	Coeff_crude_	95%CI		p	R^2^	R^2^adj	Coeff_adj_	95%CI		p		Coeff_adj_		95%CI			p	
Education					0.044	0.026												
apprenticeship	1.95	[-1.65,5.55]		0.283			3.17	[-0.00,6.35]		0.05		2.95		[-0.11,6.01]			0.059	
primary	0.00	--		--			0.00	--		--		0.00						
secondary	-1.00	[-5.00,2.99]		0.617			-2.67	[-6.28,0.95]		0.144		-2.54		[-6.03,0.94]			0.149	
specialized	1.28	[-4.55,7.11]		0.661			-1.48	[-6.90,3.94]		0.586		-0.38		[-5.66,4.90]			0.886	
university	-2.72	[-9.77,4.34]		0.443			1.13	[-5.16,7.41]		0.72		1.86		[-4.25,7.96]			0.544	
Previous inpatient stays	0.15	[0.03,0.27]		**0.017**														
HCR-20	0.18	[0.03,0.34]		**0.023**	0.086	0.070	0.19	[0.03,0.34]		**0.017**								
Historical	0.31	[0.04,0.58]		**0.024**	0.085	0.069						0.01		[-0.32,0.34]			0.948	
Clinical	0.72	[0.30,1.16]		**0.001**	0.165	0.151						0.70		[0.18,1.22]			**0.009**	
Risk	0.16	[-0.41,0.73]		**0.574**	0.006	0.000												
LOSlog	1.40	[0.12,2.68]		**0.033**	0.076	0.060	1.75	[0.53,2.96]		**0.006**		1.47		[0.28,2.66]			**0.017**	
Pretrial detention	-2.86	[-5.25,-0.48]		**0.020**	0.09	0.075	-2.50	[-4.80,-0.19]		**0.034**		-2.41		[-4.69,-0.12]			**0.039**	
Male sex	-2.61	[-5.77,0.54]		0.103	0.045	0.029	-2.63	[-5.57,0.31]		0.078		-2.15		[-5.06,-0.24]			0.054	

Only education and diagnosis of borderline personality disorder were related to the LoS (logarithm) in the present study. In univariate models, secondary education was associated with longer LoS whereas borderline personality disorder was the only diagnosis to be negatively associated with this variable. In multivariable modes, these parameters remained significant predictors of the LoS and explained 12.6% of its variance (Table 3).

**Table 3:** Results of univariate (Coeff. unadjusted) and multiple (Coeff. adjusted OR) linear regression associated with the logarithm of length of stay. N = 60. R^2^ : coefficient of determination; R^2^ adj: adjusted R^2^

Characteristics	Coeff crude	95% CI	p-value	R^2^	R^2^adj	Coeff adjusted	95% CI	p-value	R^2^	R^2^adj
Education				0.092	0.026					
apprenticeship	-0.15	[-0.84, 0.54]	0.659			-0.23	[-0.88, 0.43]	0.493		
primary	0.00	--	--			0.00	--	--		
secondary	0.83	[0.06, 1.59]	**0.035**			0.89	[0.16, 1.62]	**0.018**		
specialized	0.06	[-1.06, 1.18]	0.913			0.16	[-0.90, 1.22]	0.762		
university	-0.24	[-1.59, 1.12]	0.726			-0.40	[-1.69, 0.89]	0.539		
Borderline personality	-0.68	[-1.27, -0.09]	**0.024**	0.085	0.069	-0.78	[-1.36, -0.20]	**0.009**		
									0.200	0.126

## Discussion

Our data make it possible to determine the clinical and criminological profile as well as patterns of outcome including symptom evolution and length of stay in an acute care setting specialized in forensic psychiatry in Geneva. Compared to those on sentence execution, cases on pre-trial detention are less symptomatic at admission and show lower changes of HONOS scores at discharge and lower levels of psychopathy, and benefit from less protective factors. Our data reveal a better outcome for patients with higher risk for violent behaviours according to the HCR-20, sentence execution and previous history of psychiatric inpatient care. Importantly, the clinical diagnosis is not a significant determinant of the outcome in acute psychiatric care of detained persons. Moreover, they show that the LoS is independent on the criminological and sociodemographic factors and was significantly lower only in cases with borderline personality disorder.

Changes in the severity of acute symptoms were frequently used to assess the clinical evolution in care settings of general psychiatry. Greater severity of symptoms at admission was usually related to better outcomes [39–41]. Compulsory admission, mood and anxiety disorders, absence of personality disorders and substance use disorders, but also single stays were all associated with more favourable clinical evolutions [39, 41–44]. Controlling for baseline HONOS-secure/HONOS scores, our findings show that higher HCR-20 scores, longer LoS and number of previous hospitalizations were positively associated with HONOS-secure/HONOS score changes indicating that patients with more severe risk for violence, familiar with psychiatric care prior to incarceration are more susceptible to benefit from longer stays in acute care forensic units. A strong positive association was found between the score of clinical and historical but nor risk HCR-20 items and improvement during hospital stay. Importantly, this finding is diagnosis-independent and cannot thus be explained by the positive effect of hospitalization for detained persons with long-lasting psychosis. In contrast, pre-trial detention is associated with lower change of HONOS-secure/HONOS scores upon discharge pointing to the increased vulnerability of this population that accumulates severe stress due to the uncertainty of the final sentence. Most importantly and unlike that reported in general psychiatry settings, the type of diagnosis has no independent effect on HONOS secure/HONOS score evolution in our series. Taking together these results suggest that the impact of the acute care in forensic psychiatry settings depends more on criminological profile, previous exposure to psychiatric care and legal status than clinical diagnosis per se. The association of higher HCR-20 clinical items score and sentenced status with higher delta HONOS scores persisted in multivariable models further supporting the relevance of these factors in predicting better clinical outcome in this care setting.

We also found significant differences in the determinants of LoS in our context compared to both long term stays in medium and high security hospitals but also acute care units in general psychiatry. Both criminological factors such as age at first incarceration and type of offense were consistently associated with LoS in medium and high security hospitals. Not surprisingly, older age at first admission, violent crimes and severe offenses (mainly sexual assaults) were related to longer LoS in these settings [6, 8, 10, 12, 45–47]. The only clinical determinant of longer LoS was the presence of psychosis [8, 10, 12]. In general psychiatry, the number of previous hospitalisations, diagnosis of schizophrenia or mood disorders, and female gender determine longer LoS in acute care whereas the opposite was true for substance use and borderline personality disorders [48, 49]. Controlling for all of these candidate predictors, and besides the effect of secondary level of education that was independently associated with longer LoS possibly reflecting a better acceptance of care, the present study shows that only the diagnosis of borderline personality disorder is associated with shorter duration of stay in forensic acute care settings. This sole parameter explained 12.6% of the LoS variance, a modest but still significant percentage if one considers the significant number of clinical, demographic and environmental factors that impact on this variable. As in general psychiatry, the rapid regression of disruptive behaviour and emotional disturbances may allow for a rapid discharge even with modest changes in HONOS secure/HONOS scores. Of importance, although the risk of violence, assessed with the HCR-20 score, impacts on the clinical outcome it seems unrelated to the LoS. This may be explained by the fact that in contrast to medium and high security hospitals where the discharge implies the transition to more permissive settings (psychiatric hospital, residential care), the end of stay in our unit was followed by a return to prison. In this context, the decision of discharge may be taken on the basis of the clinical evolution solely since it does not imply an increased risk of recidivism. In the same line and in contrast to both medium and high security hospitals as well as acute care in general psychiatry, the diagnosis of psychosis, present in 50% of our sample, does not lead to increased LoS. This may be also due to the absence of immediate consequences of the discharge made in prison on risk of recidivism and psychosocial repercussions in the community. Unlike that reported in general psychiatry settings, the diagnosis of lifetime substance use disorders, that was identified in 70% of our sample, was not associated with shorter LoS. Without the legal constraints imposed by the incarceration, a significant proportion of patients with comorbid substance use disorders interrupt their stay due to craving and intolerance to the hospital rules [50, 51].

### Strengths and limitations

Strengths of the present study is admission of all cases in the same unit of acute psychiatric care in prison that decreases the variability in the admission criteria, multidimensional characterization of the sample including sociodemographic, clinical and criminological parameters, and use of multivariable models controlling for the variables known to impact on clinical outcome and LoS in both general psychiatry and long term forensic psychiatry settings. Several limitations should, however, be mentioned. Clinical diagnosis was carried out by two independent clinicians blinded to the aim of the study. Standardized diagnostic questionnaires were not used in order to be close to a real-life situation. Moreover, it has to be mentioned that criminal records included prior convictions in Switzerland and countries of the European Union. On the contrary, convictions in other countries (including the native) were assessed solely on the basis of self-reports during the hospital stay. In this situation, we cannot thus exclude a declaration bias that could affect the quality of this variable. In the same line, the assessment of previous inpatient stays outside the Geneva county was also made by self-report and could be biased. The difference in time spent in prison may impact per se on the clinical outcome. To address partly this limitation, our randomization process prevents the overrepresentation of cases with repeated admissions during the period of reference. The negative results regarding bipolar disorder, substance use disorders and antisocial personality should be interpreted with caution given the limited sample. This was also the case for some sociodemographic variables such as university education. Last but not least, these observations concern a specialized unit of forensic psychiatry located in prison and not in a psychiatric hospital. These latter may be radically different in the absence of prison staff that implies an *a priori* selection of cases with better criminological profiles. Future studies in larger samples using standardized assessment of clinical diagnosis, detailed assessment of previous convictions, and inclusion of forensic psychiatry units outside the prison are needed to explore the determinants of clinical outcome and LoS in acute care forensic psychiatry settings.

## Conclusions

From a clinical viewpoint, our results suggest that the use of acute wards specialized in forensic psychiatry could be mainly useful for patients with prior inpatient care experience, and higher violence risk during sentence execution. Importantly, these independent variables explain more than 25% of the delta HONOS variance, a quite substantial percentage given the complexity of factors that impact on this clinical parameter. In contrast, they seem to be less performant for persons in pre-trial detention that could benefit from less restrictive clinical settings. However, one should keep in mind that in our sample, pre-trial patients displayed less frequently psychotic disorders that are known to increase the risk of violence in clinical settings. The present findings also indicate that, unlike both general psychiatry and long-term forensic psychiatry settings, in the context of acute care, the forensic parameters are more pertinent than the clinical diagnosis in the prediction of outcome measures.

## Data Availability

Data and Material are available upon request from corresponding author. The raw data supporting the conclusions of this article will be made available by the corresponding author, without undue reservation.
